# The SAM Domain of Human TEL2 Can Abrogate Transcriptional Output from TEL1 (ETV-6) and ETS1/ETS2

**DOI:** 10.1371/journal.pone.0037151

**Published:** 2012-05-17

**Authors:** Pavithra Vivekanand, Ilaria Rebay

**Affiliations:** Ben May Department for Cancer Research, University of Chicago, Chicago, Illinois, United States of America; University of Massachusetts Medical School, United States of America

## Abstract

Regulation of gene expression downstream of the Receptor Tyrosine Kinase signaling pathway in *Drosophila* relies on a transcriptional effector network featuring two conserved Ets family proteins, Yan and Pointed, known as TEL1 (ETV6) and ETS1/ETS2, respectively, in mammals. As in *Drosophila*, both TEL1 and ETS1/ETS2 operate as Ras pathway transcriptional effectors and misregulated activity of either factor has been implicated in many human leukemias and solid tumors. Providing essential regulation to the *Drosophila* network, direct interactions with the SAM domain protein Mae attenuate both Yan-mediated repression and PointedP2-mediated transcriptional activation. Given the critical contributions of Mae to the *Drosophila* circuitry, we investigated whether the human Ets factors TEL1 and ETS1/ETS2 could be subject to analogous regulation. Here we demonstrate that the SAM domain of human TEL2 can inhibit the transcriptional activities of ETS1/2 and TEL1. *Drosophila* Mae can also attenuate human ETS1/ETS2 function, suggesting there could be cross-species conservation of underlying mechanism. In contrast, Mae is not an effective inhibitor of TEL1, suggesting the mode of TEL2SAM-mediated inhibition of TEL1 may be distinct from how *Drosophila* Mae antagonizes Yan. Together our results reveal both further similarities and new differences between the mammalian and *Drosophila* networks and more broadly suggest that SAM domain-mediated interactions could provide an effective mechanism for modulating output from the TEL1 and ETS1/2 oncogenes.

## Introduction

The evolutionarily conserved Receptor Tyrosine Kinase (RTK)/Ras/mitogen-activated protein kinase (MAPK) signal transduction cascade regulates such diverse processes as cell fate specification, proliferation, differentiation and survival [1]. Consistent with its essential roles in development, misregulation at any step in the RTK pathway, from the receptor down to the nuclear transcriptional effectors, contributes to the initiation and progression of a broad spectrum of human malignancies [2].

**Figure 1:**
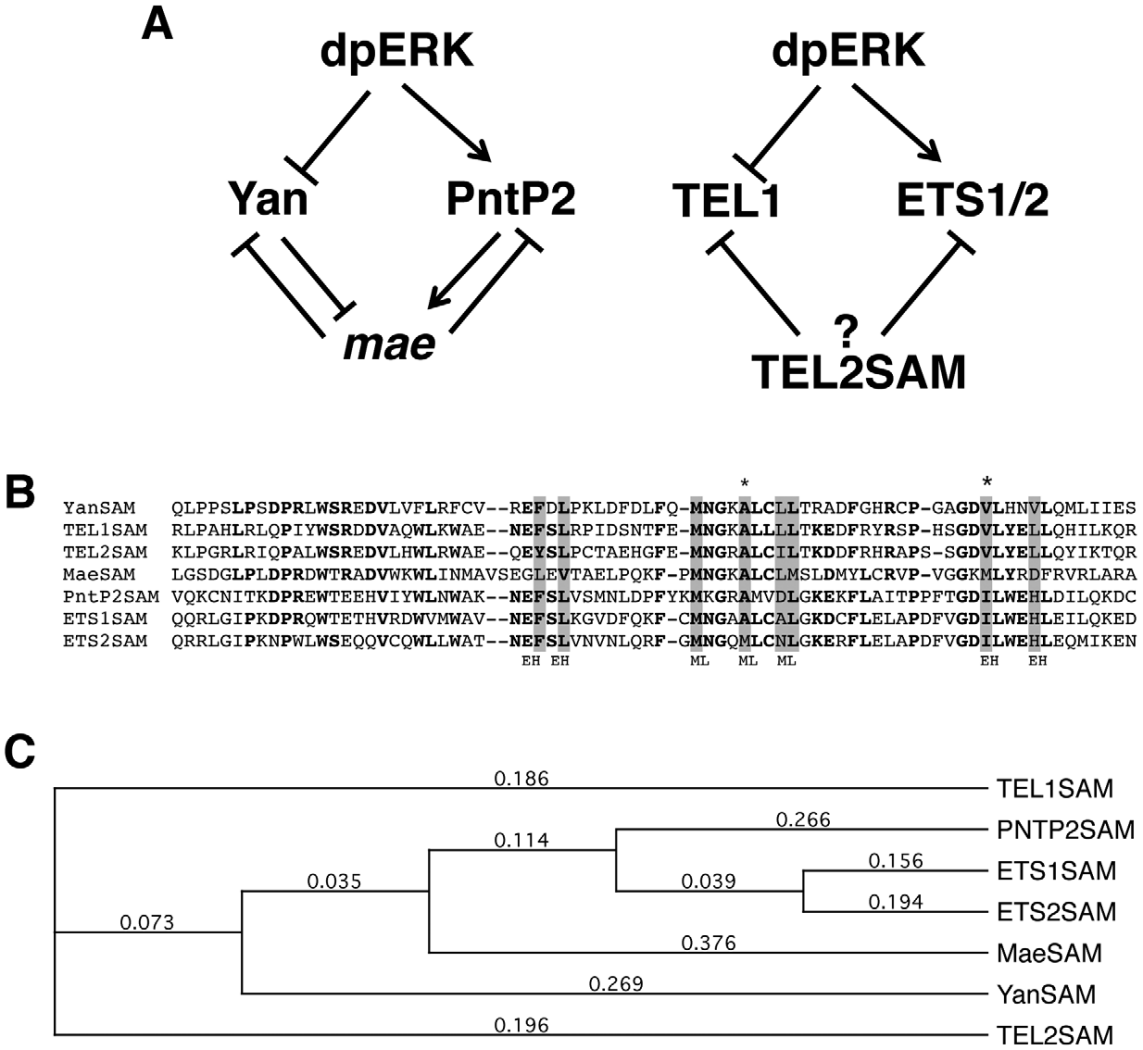
Conservation of the mammalian and Drosophila Ets networks. (A) Schematic representation of the *Drosophila* and Mammalian Ets Networks. Activated MAPK (dpERK) phosphorylates Yan (TEL1) and PntP2 (ETS1/2) to inhibit transcriptional repression of target genes by Yan and to potentiate transcriptional activation by PntP2 respectively. Mae negatively regulates Yan and PntP2 to modulate signaling by the RTK network. Similarly, TEL2SAM negatively regulates the transcriptional activity of the vertebrate orthologs TEL1 and ETS1/2. (B) Sequence alignment of the SAM domains of Yan, TEL1, TEL2, Mae, PntP2, ETS1 and ETS2. Amino acids that are identical in at least four of the seven proteins are in bold, grey boxes highlight critical residues that mediate EH-ML surface interactions, and the asterisks indicate the specific residues mutated in the TEL2SAM^EHmut^ and TEL2SAM^MLmut^ constructs. (C) Dendrogram analysis using the sequences in (B) shows the phylogenetic relationships of the SAM domains.

Work from multiple laboratories has defined a critical RTK pathway transcriptional effector circuit in *Drosophila* in which the four core components, MAPK, Yan, PointedP2 (PntP2) and Mae, are interconnected via multiple levels of transcriptional regulation, protein-protein interactions, and post-translational modifications (Figure 1A; reviewed in [3]). At the top of this signaling module, that we will refer to as the E twenty-six (Ets) network, activated MAPK (or dpERK, dually phosphorylated extracellular signal-regulated kinase) directly phosphorylates a pair of functionally antagonistic Ets family transcription factors, Yan and PntP2 [4], [5], [6]. This attenuates the repressor function of Yan and stimulates the trans-activation ability of PntP2, thereby effecting a switch such that target genes previously bound and repressed by Yan are activated by PntP2. A Sterila Alpha Motif (SAM) domain containing protein named Modulator of activity of Ets (Mae), itself a direct transcriptional target of both Yan and PntP2, provides dual positive and negative feedback regulation by binding directly to the SAM domains of both Yan and PntP2 and inhibiting their respective transcriptional activities [7], [8], [9], [10].

Of the four nodes within the *Drosophila* Ets Network, three have been identified in mammals: ERK, the Yan ortholog TEL1 (Translocation Ets Leukemia; also referred to as ETV-6, ETS Variant 6) and the PntP2 orthologs ETS1 and ETS2. Like its *Drosophila* counterpart Yan, TEL1 is a transcriptional repressor whose function is negatively regulated by ERK-mediated phosphorylation [11], [12], while ETS1 and ETS2, like PntP2, are activators that require stimulation by dpERK (Figure 1A) [13]. Reflecting their normal developmental roles in regulating proliferation and differentiation in a variety of tissues, misregulated activity of TEL1 and ETS1/2 provides an oncogenic driving force for a variety of solid tumors and leukemias [14]. The high degree of conservation of this signaling module across species is underscored further by the observation that expression of human ETS1/2 in *Drosophila* can partially rescue *pnt* mutant phenotypes [15].

In addition to the Ets family DNA binding motif, Yan/TEL1 and PntP2/ETS1/ETS2 all carry a second conserved domain, the sterile alpha motif (SAM; Figure 1B, C). SAM domains mediate both homotypic and heterotypic protein-protein interactions, and are found in a broad spectrum of proteins including a subset of Ets family members [16], [17], [18], [19]. Both Yan and TEL1 oligomerize via their N-terminal SAM domains [11], [16], [17]. This interaction is required for transcriptional repression as introduction of missense mutations that restrict the protein to a monomeric form abrogates repressor activity [11], [16], [17], [20]. Although Yan/TEL1 monomers retain DNA binding ability, a recent study showed that dimerization can confer cooperativity [21]. In the case of TEL1, chromosomal translocations that fuse the N-terminal SAM containing region to either protein tyrosine kinases such as PDGFR (platelet derived growth factor receptor) and Abelson, or to transcription factors such as AML1 (acute myeloid leukemia) have been associated with a number of hematopoietic malignancies [14]. The homotypic interaction ability of the TEL1 SAM domain is thought to contribute to the pathogenesis of these chimeric fusion proteins [22]. Mae, a monomeric SAM domain protein, abrogates Yan-mediated transcriptional repression via a heterotypic MaeSAM-YanSAM interaction that disrupts oligomerization in vitro [7], [8], [17]. Although PntP2/ETS1/ETS2 do not self-associate via their SAM domains, heterologous SAM-SAM interactions with Mae abrogate PntP2 activity [7], [8], [9], [10], [23].

Despite the multiple layers of critical regulation that Mae contributes to the *Drosophila* Ets network, and the extensive functional conservation between Yan/TEL1 and PntP2/ETS1/2, to date no mammalian Mae equivalent has been identified. To address this gap, we investigated whether comparable SAM-mediated interactions could influence the vertebrate Ets network. Here we demonstrate that the SAM domain from the human Ets family member TEL2 can antagonize the transcriptional activities both ETS1/ETS2 and TEL1. Further, *Drosophila* Mae can effectively antagonize human ETS1/2, suggesting cross-species mechanistic conservation. However Mae is not an effective inhibitor of TEL1, nor is human TEL2 a strong antagonist of *Drosophila* Yan. This suggests that although both Mae-Yan and TEL2-TEL1 interactions can abrogate transcriptional repression activity, the underlying mechanisms may be distinct. More broadly, our results suggest that further exploration of SAM-mediated inhibitory interactions could lead to development of therapeutic reagents that attenuate the oncogenic activities of human Ets proteins.

## Materials and Methods

### Plasmids

For expression in *Drosophila* cultured cells ETS1, ETS2 and TEL2 were PCR amplified using 5′ETS1-KpnI aagggtaccaaggcggccgtcgat, 3′ ETS1-SacI aagagctcctagtcagcatccggctt, 5′ETS2-Sal1 gaggtcgaccaatgactttggaatc, 3′ETS2-Not1 aagcggccgctcagtcttctgtatcaggc, 5′TEL2-Kpn1 aagggtacccaggagggagaattgg, 3′TEL2-Sal1 gaggtcgactcacggagagatttctggc from pCMVTag2a-ETS1, pCMVTag2a-ETS2, pMSCV-FlagTEL2-I-GFP plasmids respectively and cloned into pRmHa3-Flag vector. The argos-luciferase reporter was generated by PCR amplification using 5′ arg-KpnI ggggtacctaacggtgatgtctttg and 3′arg-NdeI gcaattccatatgataccggaagtccggaagtg from genomic DNA and cloned upstream of the luciferase ORF. ETS1, ETS2 expression plasmids, MMP9 and dEtsluciferase reporter plasmids were provided by Dr. Barbara Graves and TEL1 and TEL2 constructs by Dr. Gerard Grosveld. For expression in HeLa cells, TEL1 and TEL2 were subcloned as EcoRI fragements from pMSV-ttTel and pMSCV-FlagTEL2-I-GFP respectively into pCDNA3.1. TEL2SAM (1–117aa) was generated by PCR amplification using 3′TEL2-XhoI (117aa) catctcgagttaccgctgggtcttgatgt and cloned into pCDNA3.1. TEL2SAM^MLmut^ and TEL2SAM^EHmut^ were generated using site directed mutagenesis.

### Transfection and Transcription assays

*Drosophila* cultured S2 cells obtained from the *Drosophila* Genomics Resource Center were grown in Gibco Sf-900 serum-free medium (Invitrogen) and transfected using DDAB with 1.0 ug of argos-luciferase reporter, 2.0 µg of expression plasmids and 0.5 µg of pActLacZ to normalize for transfection efficiency. HeLa cells obtained from the ATCC (CCL-2) were cultured in MEM supplemented with 10% fetal bovine serum and transfected using lipofectamine with 0.5 µg of MMP9-luciferase and dEts-luciferase reporters, 1.0 µg of expression plasmids and 200 ng of Renilla luciferase to normalize for transfection efficiency. To analyze TEL1 repression HeLa cells were transfected with 2.0 µg of E74tkLuciferase, 200 ng of Renilla luciferase and 1.0 µg of the respective expression plasmids. Transcription assays were performed using the luciferase and Galacto star kits (Tropix) for *Drosophila* cultured cells and the Dual luciferase assay system (Promega) for HeLa cells.

### Co-immunoprecipitations

*Drosophila* cultured cells were lysed by sonication in lysis buffer (50 mM Tris pH 7.5, 100 mM NaCl, 2 mM EDTA, 2 mM EGTA, 1% NP40). Clarified lysates were incubated with 20.0 ul of Flag conjugated agarose beads for 3 hours at 4°C, washed 3×5 min in lysis buffer and run on 8 or 12% SDS polyacrylamide gels. HeLa cells were lysed by sonication in lysis buffer (50 mM Tris pH 7.5, 100 mM NaCl, 1 mM EDTA, 1% Triton-X). Lysates were incubated with 15.0 ul Myc agarose beads overnight at 4°C, followed by 3×5 min washes in wash buffer (50 mM Tris pH 7.5, 100 mM NaCl, 1 mM EDTA, 0.25% Triton-X) and run on 8 or 15% SDS polyacrylamide gels. The following antibodies were used for western blotting, Rbα Flag 1∶5000 (Sigma), mα Myc 1∶1000 (Santa Cruz Biotechnology) and Rbα HA 1∶5000 (Rockland).

## Results and Discussion

### TEL2SAM can inhibit transcriptional repression by TEL1

Considering the structural and functional similarities between the Yan and TEL1 repressors, together with the pivotal regulation provided by Mae with respect to Yan, we speculated that a Mae-like protein might similarly regulate TEL1 activity. Another Ets family member, human TEL2, provided an intriguing candidate as it has been shown to interact with and antagonize the ability of TEL1 to inhibit Ras induced cellular transformation, although the underlying mechanism has not been elucidated [24], [25]. Of further interest is a splice isoform, TEL2a, that is predicted to encode a protein consisting of only the N-terminal SAM domain [26], which would be structurally quite similar to *Drosophila* Mae. We therefore postulated that human TEL2 might provide a functional counterpart to *Drosophila* Mae by interacting with and inhibiting TEL1-mediated transcriptional repression.

**Figure 2:**
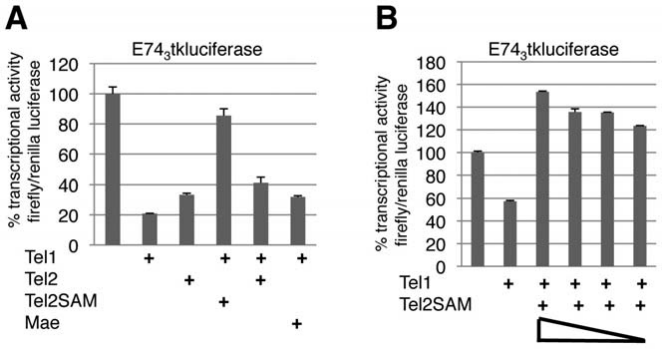
TEL2SAM inhibits transcriptional repression by TEL1 of the E74tkluciferase reporter. (A) Repression by TEL1 is suppressed by TEL2SAM but *Drosophila* Mae has only a weak effect. The full-length TEL2 alone lane derives from an independent experiment in which repression by TEL1 was almost identical to that shown here. Using the TEL1 alone values to normalize between experiments, the % transcriptional activity for TEL2 was adjusted by a factor of 0.82. (B) TEL2SAM effectively inhibits repression by TEL1 at decreasing TEL2SAM concentrations. The ratio of TEL2SAM DNA to TEL1 DNA that was used for titration ranged from 2∶1 to 0.25∶1.

To address this question we performed transcription assays in transiently transfected HeLa cells and examined the ability of TEL1 to repress the E74_3_tkluciferase reporter [11] in the presence and absence of either full-length TEL2 or TEL2SAM, a construct designed to mimic both TEL2a and Mae. Like TEL1, both TEL2 and TEL2SAM were predominantly nuclear as judged by indirect immunofluorescence, and western blot analysis confirmed expression of products of the expected size (data not shown). Expression of TEL1 or TEL2 alone resulted in respective five-fold and three-fold repression of the E74_3_tkluciferase reporter, while TEL2SAM alone, which lacks a DNA binding domain, had no effect (Figure 2A and data not shown). Co-expression of TEL2SAM almost completely attenuated transcriptional repression by TEL1, while coexpression of full-length TEL2 with TEL1 resulted in a level of repression (2.5-fold) comparable to that of TEL2 alone (Figure 2A). Because interpreting the results with full length TEL2 is complicated by the fact that TEL2′s intrinsic repression ability could mask the effects of direct SAM domain-mediated interference with TEL1 function, all subsequent experiments were performed with the TEL2SAM construct.

Because the TEL2a isoform has been reported to be expressed at low levels [26], we reduced the levels of TEL2SAM in our assay system to more accurately reflect the presumed physiological situation in which TEL2a would be present in substoichiometric ratio to TEL1. Our results revealed effective inhibition of TEL1-mediated repression even when the amount of TEL2SAM DNA used for transfection was 0.25 times that used for TEL1 (Figure 2B). This suggests the functional interactions observed in transfected cells should be possible in situ, and emphasizes the importance of developing suitable reagents to explore interactions between endogenous TEL2a and TEL1 in normal and malignant tissues.

### Distinct mechanisms of TEL2SAM inhibition of TEL1 and Mae inhibition of Yan

**Figure 3:**
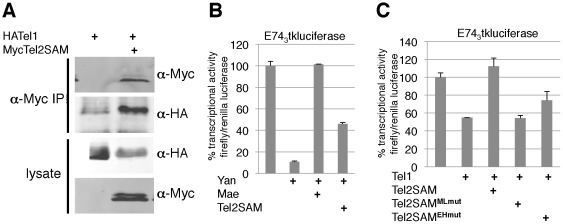
Inhibition of TEL1 repression by TEL2SAM is alleviated by mutations that prevent SAM domain-polymerization. (A) Communoprecipitation of myc-TEL2SAM with HA-TEL1 from cotransfected HeLa cells (lane 2) but not from cells transfected with HA-TEL1 alone (lane 1). Top and bottom panels were from the same gel, as were the middle two panels. (B) TEL2SAM can inhibit transcriptional repression by Yan, although not as effectively as Mae. (C) Repression of the E74tkluciferase reporter by TEL1 is suppressed by TEL2SAM but not by TEL2SAM^EHmut^ or TEL2SAM^MLmut^.

The ability of TEL2SAM to inhibit TEL1-mediated transcriptional repression suggests TEL2SAM could provide a functional counterpart to *Drosophila* Mae. Consistent with this hypothesis, co-immunoprecipitation experiments revealed association of the TEL2SAM and TEL1 (Figure 3A). This suggests that abrogation of TEL1-mediated repression results from heterodimeric TEL1-TEL2SAM interactions, just as SAM-mediated Yan-Mae complexes have been shown to attenuate Yan repression activity.

To determine the extent of cross-species conservation, we asked whether Mae could inhibit TEL1 and whether TEL2SAM could inhibit Yan. Unexpectedly, *Drosophila* Mae only modestly attenuated TEL1-mediated repression (Figure 2A). Mae was not simply misfolded and inactive in the HeLa cell environment because it could completely attenuate Yan-mediated repression of the same reporter (Figure 3B). TEL2SAM was able to inhibit Yan-mediated repression, although it was less effective than Mae (Figure 3B). These results demonstrate that the SAM domains of both human TEL2 and *Drosophila* Mae share the ability to attenuate TEL1/Yan-mediated repression. However the inability of Mae to antagonize TEL1 and the reduced ability of TEL2SAM to antagonize Yan suggest the underlying mechanisms could be different.

Mechanistically, because the SAM domain of TEL2 can itself oligomerize, it has been proposed that it might form a copolymer with TEL1 [16], [19], [27]. This is in contrast to *Drosophila* Mae, where replacement of one of the key hydrophobic residues in the EH surface with a negatively charged aspartate (Figure 1B) prevents the EH-ML interaction and limits the protein to a monomeric state. Mae retains a functional ML surface and so can form heterodimers, but not hetero-oligomers, with Yan. By “capping” the extent of Yan polymerization, Mae thus attenuates Yan's repression activity [27].

Because *Drosophila* Mae was an ineffective TEL1 antagonist, we hypothesized that TEL1-TEL2SAM heteropolymerization might contribute to abrogation of TEL1-mediated repression. Consistent with this interpretation, mutation of a conserved residue in either the EH or ML domain of TEL2SAM that is predicted to interfere with SAM domain mediated interactions [17], produced a TEL2SAM protein that was unable to inhibit repression by TEL1 (Figure 3C). Together our results demonstrate that SAM-mediated interactions with TEL2 can antagonize TEL1-mediated repression activity, but that the underlying mechanism appears distinct from how Mae antagonizes Yan activity. Further biochemical analysis will be required to reveal the precise mechanisms and stoichiometry of TEL1-TEL2 and TEL1-TEL2SAM interactions and to explain why TEL2-TEL1 heteropolymers should be less effective repressors than TEL1 homopolymers.

Could other SAM domain proteins also negatively regulate TEL1 function? The fact that the *TEL2* gene is deleted in rodents, and even in humans is unlikely to be universally coexpressed with *TEL1* [26], suggests the likelihood of functionally redundant mechanisms. Thus a broader investigation of SAM-mediated inhibition of TEL1 activity may be warranted. While such regulation need not derive solely from other Ets family members, SAM-mediated protein-protein interactions between Tel-1 and Fli-1 have been reported [28]. Two charged residues in the ML region of the Fli-1SAM domain are predicted to interfere with its self-association, consistent with its placement in the subset of monomeric Ets family members. However, while Tel-1 has been shown to interfere with the ability of Fli-1 to function as a transcriptional activator [28], whether Fli-1 can antagonize Tel-1 mediated repression has not yet been investigated. Similarly in *Drosophila*, investigation of potential regulation of Yan via heteropolymeric interactions with other SAM containing proteins could prove fruitful.

### Negative regulation of ETS1/ETS2 by Mae and TEL2SAM

*Drosophila* Mae provides both positive and negative feedback regulation within the Ets Network [10]. Thus in addition to facilitating down-regulation of Yan-mediated repression to allow PntP2 to activate key downstream target genes, Mae attenuates the transcriptional response to RTK signaling by associating with PntP2 and blocking its MAPK docking site, thereby preventing phosphorylation-induced activation [23]. As with the Yan-Mae interaction, the PntP2-Mae interaction occurs via their respective SAM domains [8], [9], [23]. Like PntP2, mammalian ETS1 and ETS2 are nuclear effectors of RTK signaling that are activated by MAPK-mediated phosphorylation of a conserved threonine residue N-terminal to the SAM domain (Figure 1A) [4], [5], [13]. Given the structural and functional similarities between PntP2 and ETS1/2, we asked whether Mae could abrogate transcriptional activation by ETS1 and ETS2.

**Figure 4:**
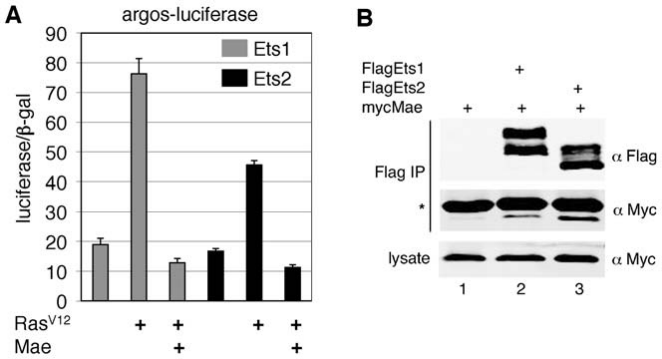
Mae suppresses transcriptional activation by ETS1/2 in *Drosophila* S2 cells. (A) Activation of the argos-luciferase reporter by ETS1/2 is enhanced by expression of Ras^V12^ and inhibited by Mae. (B) Myc-Mae coimmunoprecipitated with Flag-ETS1/2 from lysates of cotransfected *Drosophila* S2 cells cotransfected (lanes 2 and 3) but not from lysates of cells transfected with Myc-Mae alone (lane 1). Myc-Mae runs below the IgG light chain (strong band marked with asterisk). Flag-ETS1/2 run as doublets.

We addressed this question first by following transcriptional reporter expression in transfected *Drosophila* S2 cells, an assay system that we and others have exploited previously to elucidate Mae function with respect to PntP2 [8], [9], [23]. Using argos-luciferase, a reporter construct derived from a Yan and PntP2 responsive regulatory element from the transcriptional target *argos* [29], we analyzed the ability of ETS1 and ETS2 to activate transcription in S2 cells. Further demonstrating their functional similarity to PntP2 [5], [8], [15], both ETS1 and ETS2 activated the reporter in a manner that was enhanced by co-expression of constitutively active Ras (Ras^V12^) (Figure 4A). Addition of Mae effectively repressed Ras/MAPK stimulated transcriptional activation by ETS1 and ETS2 (Figure 4A) and co-immunoprecipitation experiments demonstrated that Mae could interact with both proteins (Figure 4B).

**Figure 5:**
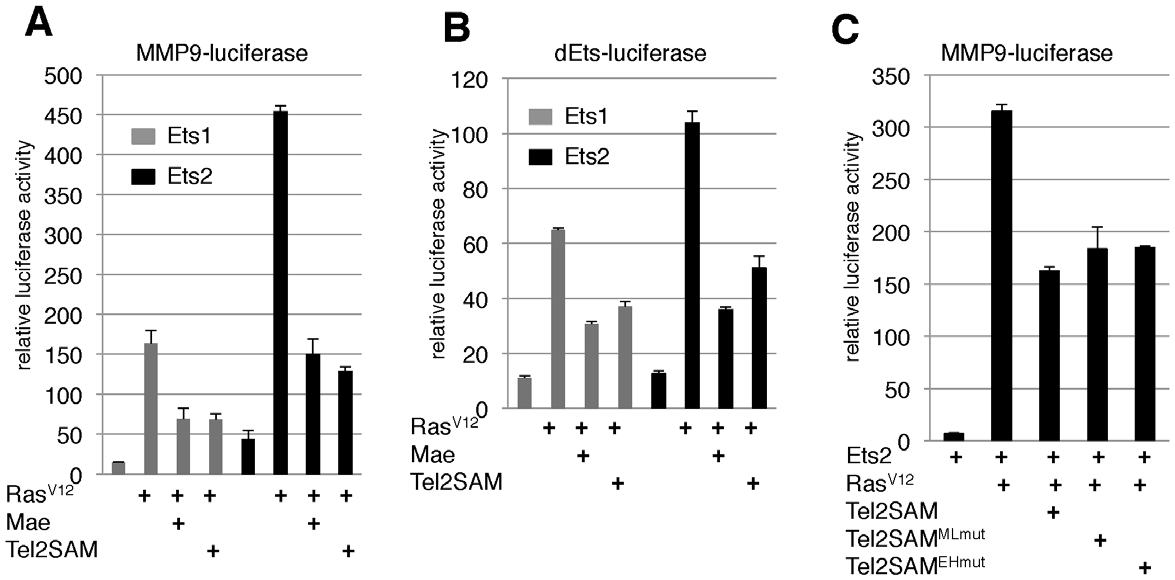
TEL2SAM inhibits transcriptional activation by ETS1/2 in HeLa cells. Ras^V12^ enhanced and Mae or TEL2SAM suppressed activation of (A) the MMP9-luciferase reporter and (B) the dEts-luciferase reporter. (C) Mutations in the EH or ML surfaces of TEL2SAM do not alter its ability to suppress ETS2.

To assess further Mae-mediated negative regulation of ETS1 and ETS2, we examined the ability of Mae to prevent transcriptional activation of native Ets target genes by performing transcription assays in transiently transfected HeLa cells. Two transcriptional reporters, MMP9-luciferase and dEts-luciferase, that contain Ras responsive elements (RRE) from the regulatory regions of the ETS1/ETS2 target genes MMP9 and MMP3, were assayed [30]. As in S2 cells, Mae effectively prevented Ras stimulated transcriptional activation by ETS1 and ETS2 (Figure 5A, 5B). Together these results suggest that the mechanism of Mae-mediated antagonism of PntP2 in *Drosophila* [8], [9], [23] could be relevant to ETS1/ETS2 regulation in mammals.

The activities and regulation of ETS1 and ETS2 were slightly different in the two cell types that were used for our analysis. In S2 cells, we observed higher levels of luciferase reporter activation by ETS1 than ETS2 (Figure 4A), whereas in HeLa cells ETS2 was a more efficient activator (Figure 5A, 5B). This might reflect a difference in the ability of Mae to interact with and negatively regulate ETS1 and ETS2. Supporting this idea, Mae was more efficiently immunoprecipitated by ETS2 than by ETS1 (Figure 4B, compare lane 3 and lane 2) even though it was expressed at comparable levels. Since Mae is expressed endogenously in S2 cells, the lower activation by ETS2 might be a result of inhibition by endogenous Mae. Also, Mae was able to negatively regulate ETS1/2 more effectively in S2 than in HeLa cells, perhaps reflecting reduced functionality of the *Drosophila* protein at the higher temperature used for culturing HeLa cells. Alternatively, endogenous ETS1/ETS2 proteins in HeLa cells could interact with Mae and limit its ability to repress function of the overexpressed constructs.

We next asked whether TEL2SAM could function analogously to Mae by analyzing the effect of TEL2SAM on ETS1/2-mediated activation of the MMP9 and dEts-luciferase reporters. TEL2SAM was able to prevent Ras stimulated transcriptional activation of both reporters by ETS1 and ETS2 (Figure 5A,B). Furthermore, TEL2SAM oligomerization was not required, as EH or ML surface mutations did not compromise activity (Figure 5C). Thus TEL2SAM behaves similarly to *Drosophila* Mae with respect to antagonism of ETS1/2 transcriptional activity. Future biochemical analyses will be needed to determine whether TEL2SAM negatively regulates ETS1/2 function by blocking the MAPK phosphorylation site, just as has been previously shown in the Mae-PntP2 interaction [23], or whether it acts via a different mechanism.

In conclusion, our work predicts that SAM-mediated interactions are likely to modulate the activity of mammalian TEL1 and ETS1/ETS2, as has been previously shown for the *Drosophila* homologs Yan and PntP2. Because misregulated activity of both TEL1 and ETS1/2 provides an oncogenic driving force for a variety of solid tumors and leukemias [14], the ability of TEL2SAM to antagonize ETS1/2 and TEL1 function suggests that assaying the presence or absence of the TEL2a isoform might be a useful diagnostic tool for predicting which malignancies retained or lacked this putative regulatory mechanism. Ultimately, our work may provide a foundation for designing therapeutic reagents that block the oncogenic function of TEL1 and ETS1/2.
